# Comparative Analysis of Brain and Fat Body Gene Splicing Patterns in the Honey Bee, *Apis mellifera*

**DOI:** 10.1534/g3.118.200857

**Published:** 2019-02-21

**Authors:** Kavya Kannan, Molly Shook, Yang Li, Gene E. Robinson, Jian Ma

**Affiliations:** * Carl R. Woese Institute for Genomic Biology, Department of Entomology, Neuroscience Program, University of Illinois at Urbana-Champaign, Urbana, IL 61801,; † Department of Plant Biology, University of Illinois at Urbana-Champaign, Urbana, IL 61801,; ‡ Department of Bioengineering, University of Illinois at Urbana-Champaign, Urbana, IL 61801,; § Department of Entomology, University of Illinois at Urbana-Champaign, Urbana, IL 61801,; ** Neuroscience Program, University of Illinois at Urbana-Champaign, Urbana, IL 61801,; †† Computational Biology Department, School of Computer Science, Carnegie Mellon University, Pittsburgh, PA 15213

**Keywords:** Honey bee, alternative splicing, RNA-seq, transcriptome, comparative genomics

## Abstract

RNA-seq has proven to be a powerful tool to unravel various aspects of the transcriptome, especially the quantification of alternative splicing (AS) that leads to isoform diversity. The honey bee (*Apis mellifera*) is an important model organism for studying the molecular underpinnings of behavioral plasticity and social behavior, and recent RNA-seq studies of honey bees have revealed AS patterns and their regulation by DNA methylation. However, tissue-specific AS patterns have not been fully explored. In this paper, we characterized AS patterns in two different honey bee tissue types, and also explored their conservation and regulation. We used the RNA-seq data from brain and fat body to improve the existing models of honey bee genes and identified tissue-specific AS patterns. We found that AS genes show high conservation between honey bee and *Drosophila melanogaster*. We also confirmed and extended previous findings of a correlation between gene body DNA methylation and AS patterns, providing further support for the role of DNA methylation in regulating AS. In addition, our analysis suggests distinct functional roles for tissue-specific alternatively spliced genes. Taken together, our work provides new insights into the conservation and dynamics of AS patterns across different tissue types.

Due to the development of high-throughput sequencing technologies, RNA sequencing (RNA-seq) has become the most widely used method to study transcriptomes (Wang *et al.* 2009). A major advantage of RNA-seq, as compared to microarray analysis, is that RNA-seq can detect both novel transcripts and existing annotations (Roberts *et al.* 2011; Sudmant *et al.* 2015; eGTEx Project 2017; Reyes and Huber 2018), in addition to higher accuracy in quantifying expression levels of transcripts (Fu *et al.* 2009). One application of RNA-seq that has dramatically enhanced our knowledge of the complexity of the transcriptome is the detection of alternative splicing (AS) events, which give rise to different isoforms and are key to understanding protein diversity (Guttman *et al.* 2010; Trapnell *et al.* 2010; Li *et al.* 2011).

The honey bee (*Apis mellifera*) is an important model organism to understand the gene regulatory mechanisms involved in behavior (Honeybee Genome Sequencing Consortium 2006; Menzel 2012; Zayed and Robinson 2012). Previous studies have used microarrays and RNA-seq to characterize honey bee transcriptomes and study differentially expressed genes in the brain and other tissues (Whitfield *et al.* 2003; Ament *et al.* 2012). In a recent study, RNA-seq data (Li-Byarlay *et al.* 2013) was utilized to investigate the effects of gene body methylation on gene splicing. Knock down of DNMT3 (DNA *methyl-transferase* 3) using RNA interference caused widespread and diverse changes in AS in fat body tissue. An RNAi-induced ca. 21% decrease in gene body methylation resulted mostly in changes in Exon Skipping (ES) and Intron Retention (IR) (Li-Byarlay *et al.* 2013). However, tissue-specific AS patterns in honey bees have not been systematically characterized and quantified.

Here we utilize published RNA-seq datasets to quantify and compare AS events in honey bee brain and fat body. Splice junctions in fat body and brain transcriptome data were obtained with TrueSight (Li *et al.* 2013). TrueSight integrates the mapping quality of RNA-seq reads together with the coding potential of genomic sequence to predict novel splice junctions, which is particularly useful for annotating gene models and AS patterns in non-model organisms.

To perform the AS analysis, we first used TrueSight to improve the existing annotation of the honey bee genome (version Amel 4.5), which was generated using the prediction tools GLEAN and MAKER2 (Elsik *et al.* 2014), and then we performed quantitative AS pattern analysis. We found that most characteristics of AS patterns in fat body and brain transcriptomes were quite consistent with what has been previously observed in the fruit fly *Drosophila melanogaster*, with high cross-species conservation in terms of alternatively spliced genes. Our data also support previous findings of gene body methylation regulating AS patterns in honey bee (Li-Byarlay *et al.* 2013). Additionally, we found tissue specific differences in the functional enrichment of AS genes in brain and fat body. Taken together, our analysis provides new insights into the conservation and dynamics of AS in honey bees.

## Materials and Methods

### Data used in the study

Publicly available RNA-seq data for honey bee fat body and brain were obtained from Li-Byarlay *et al.* (2013) and Rittschof *et al.* (2014), respectively. TrueSight was run on all samples individually. Raw reads were mapped to Amel 4.5 genome first using Bowtie (version 0.12.8) and then TrueSight to produce an alignment file, then a gapped alignment file, and splice junctions were inferred from the gapped alignments. The junction files obtained from TrueSight were further processed with the help of Amel 4.5 gene annotations to generate various splicing patterns and some new gene models using novel splice junctions. This is further explained in the following section.

### Modifying Amel 4.5 Gene models

#### Detecting splicing patterns*:*

Splice junctions (SJs) inferred from independent TrueSight runs were clustered together. SJs with scores greater than 0.5 were retained as TrueSight SJs and were further used to improve Gene models. Exon skipping events were confirmed when novel splice sites with both splice sites known were identified among SJs. To detect an AEB event, splice junctions with only 1 known splice site were used. The original junction linking two exons (*a* ∼ *b*; *c* ∼ *d*) is *b* ∼ *c*. If there is a junction with one known splice site: $b^{\prime }∼c$, such that $b^{\prime }-b<200,b^{\prime }>a$, exon a∼b would have alternative boundary $a∼b^{\prime }.$ Strand specificity is taken into consideration for detecting AEB events. An IR event is confirmed by two criteria: (i) each base of the intron has >5x coverage from TrueSight RNA-seq alignments in our dataset; (ii) IR inclusion ratio (described under definitions) should be at least three times higher than the average honey bee intron inclusion ratio, which is 0.017. The first criterion guarantees the IR detectable by RNA-seq, and the second criterion will screen out potential false IRs caused by RNA-seq artifacts mapped onto intron regions.

#### Identifying novel exons:

Reliable “transcribed islands” were obtained by filtering best alignments from TrueSight. Boundaries for transcribed islands were obtained by following certain criteria and only those islands were retained that did not overlap with the existing Amel 4.5 model exons. New exons and splice junctions were added after inferring AS exons in already annotated splice junctions as well as from the chosen transcribed islands using a separate algorithm. The detailed procedure for gene models modification is provided in Supplementary Methods.

### Definitions used in analyzing alternative splicing

#### IR inclusion ratio:

For a retained intron (in IR) with coordinates p∼q and two adjacent exons, a∼p,q∼d, the inclusion ratio is calculated as follows:

$$IRinclusionratio=\frac{2\times Cov(p,q)}{Cov\left(a,p\right)+Cov(q,d)}$$

where, $\mathrm{Cov}\left(\text{x},\text{y}\right)=\frac{\sum_{i=x}^{y}numberofreadsmappedontoi}{y-x}$.

#### CE inclusion ratio:

For a CE with coordinates p∼q and two adjacent constitutive exons, a∼b,c∼d, CE inclusion ratio is calculated by:

$$\text{CE inclusion ratio}=\frac{(N\left(b∼p\right)+N\left(q∼c\right))/2}{(N\left(b∼p\right)+N\left(q∼c\right))/2+N\left(b∼c\right)}$$

where, N(x∼y)=number of reads mapped onto junction x∼y.

#### CpG (o/e):

CpG (o/e) is a computational metric measuring the DNA methylation on an evolutionary time scale; it is assumed that methylated cytosines are hypermutable and low CpG (o/e) value implies depletion of CpG dinucleotides during evolution and potential hyper-methylation (Gardiner-Garden and Frommer 1987). On the other hand, high CpG (o/e) would indicate a presence of hypo-methylation.

The CpG (o/e) is defined as:

$$\text{CpG }\left(\text{o}/\text{e}\right)=\frac{P_{CpG}}{P_{C}\times P_{G}}$$

where, $P_{CpG}$, $P_{C}$, and $P_{G}$ measure the frequencies of observing CpG dinucleotides, C nucleotides, and G nucleotides, respectively.

#### AEB splicing ratio:

Let us assume that for three continuous exons (a∼b,p∼q,c∼d) in the forward strand, exon p∼q has alternative acceptor splice site p′ and alternative donor splice site q′. AEB splicing ratio for acceptor sites describes the expression ratio of transcripts using minor (less frequently used) acceptor sites to transcripts using major (more frequently used) ones, is:

$$\frac{\min(N\left(b∼p\right),N(b∼p\prime ))}{\text{max}(N\left(b∼p\right),N\left(b∼p^{\prime }\right))}$$

AEB splicing ratio for the donor sites describes the expression ratio of transcripts using minor donor sites to transcripts using major ones, is:

$$\frac{\min(N\left(q∼c\right),N(q^{\prime }∼c))}{\text{max}(N\left(q∼c\right),N\left(q^{\prime }∼c\right))}$$

where N(x∼y)=number of reads mapped onto junction x∼y.

#### AEB inclusion ratio:

AEB inclusion ratio measures the inclusion ratio of alternative exon boundaries. For the same three exons listed in the last section, the AEB inclusion ratio of region $\text{min}\left(p,p^{\prime }\right)∼\text{max}(p,p\prime )$ is:

$$\frac{N(b∼min\left(p,p^{\prime }\right))}{N\left(b∼p\right)+N(b∼p\prime )}$$

$$\text{For region }min\left(q,q^{\prime }\right)∼\text{max}(q,q\prime )$$:

$$\frac{N(max\left(q,q^{\prime }\right)∼c)}{N\left(q∼c\right)+N(q\prime ∼c)}$$

#### ATE splicing ratio:

The ATE splicing ratio measures the expression ratio of minor ATEs over major ones. Note that we only consider AFEs/ALEs directly linking to the same acceptor/donor site of a constitutive exon in this analysis. The calculation is similar to the formulas for AEB splicing ratio, using minor junction mappings over major junction mappings.

#### Splice site strength:

To calculate strength of donor and acceptor splice sites, an algorithm based on Maximum Entropy Principle (MEP) for modeling of short sequence motifs was used (Yeo and Burge 2004). The algorithms calculates how likely it is for a given region to be a true splice site based on the nucleotides surrounding the splice site. Hence, the higher the strength the more likely that region is involved in splicing.

### Gene annotations and pathway analysis

We derived Gene Ontology assignments for honey bee using protein family annotations for *Drosophila* from the database PANTHER (Mi *et al.* 2013b). Only those Gene Ontology assignments were chosen that have a p-value of 0.05 or lower for a statistical over-representation test in PANTHER (Mi *et al.* 2013a). Only KEGG pathways in *Drosophila* were chosen to perform pathway analysis of honey bee genes having fly orthologs (Ogata *et al.* 1999).

### Data availability

Additional file 1 consists of additional figures of AS pattern analysis in Brain as well as a table showing a comparison of AS results in TrueSight, TopHat2, and MapSplice. Additional file 2 contains the improved honeybee gene model after prediction of alternative splicing patterns using TrueSight. Additional file 3 contains all the AS patterns identified by TrueSight along with specific analysis results in terms of Drosophila orthology, methylation patterns, and functional categories of AS genes. Supplemental material available at Figshare: https://doi.org/10.25387/g3.7477232.

## Results

### Identification of novel exons to improve gene models

TrueSight was used to identify existing and novel splice junctions in honey bee RNA-seq reads from fat body and brain transcriptomes. Table S1 shows the performance of TrueSight in comparison with two well-known splice junction detection algorithms that also utilize reads spanning more than one junction, MapSplice (2.2.1) (Wang *et al.* 2010) and TopHat2 (v2.1.1) (Kim *et al.* 2013). Sensitivity and specificity were calculated for these three splice junction detection tools. Sensitivity is the fraction of ‘known introns’ to the largest number of ‘known introns’ discovered by one of the three methods and hence provides an estimate of the most exhaustive method (Li *et al.* 2013). Specificity is calculated by dividing the number of ‘both novel’ junctions over the ‘total’ number of splice junctions reported (Li *et al.* 2013). TrueSight showed slightly lower sensitivity but achieved the highest specificity in terms of identifying introns with both ends annotated, and so was used for this reason in this study. We identified 2,871 novel exons in total as a result of novel splice junctions (SJ) obtained from TrueSight. 30 of the newly identified exons were cassette exons (an exon that can be included or skipped in a transcript giving rise to transcript variants) and 864 of the newly added exons were novel terminal exons. These improved gene models gave rise to 1,880 more SJs in the honey bee genome, leading to a total of 71,203 SJs. The newly added junctions were added to the reference genome for tissue-specific AS pattern analysis. We also identified 989 novel multi-exon transcripts in intergenic regions in the Amel 4.5 gene models. This analysis significantly improved the existing gene model for more accurate and comprehensive AS pattern analysis in the honey bee. The new annotations are presented as a GFF3 annotation file (table S2) for viewing in the genome browsers.

### Characterization of alternative splicing events

We report on four major types of AS (Nilsen and Graveley 2010): (i) Intron retention (IR), in which an intron may be retained as part of a mature transcript or spliced out; (ii) exon skipping, in which a cassette exon (CE) may be included or skipped in a transcript; (iii) alternative use of splice sites (donor/acceptor), leading to alternative exon boundaries (AEB); and (iv) alternative terminal exons (ATE), in which alternative first exons (AFE) or alternative last exons (ALE) are used. Table I shows detailed numbers for each category. Table S3 lists all AS events obtained in fat body and brain based on TrueSight results and Fig S1-2 shows overlap of genes having more than one kind of AS event in fat body and brain respectively.

#### Cassette exons:

Out of 84,637 honey bee exons and 15,314 genes, TrueSight detected 1,520 (1.8%) CEs in fat body from 1,139 genes and 1,525 (1.8%) CEs in brain from 1,067 genes (Table 1). CEs could be detected at multiples of three lengths which reflect the maintenance of a series of consecutive nonoverlapping triplet codons during splicing events to preserve the reading frame. 24% of the CEs (273 CEs in brain and 366 in fat body) had multiples-of-three lengths. The average length of CEs was 178 bp in brain and 175 bp in fat body, smaller than the average length of all honey bee exons (320 bp). This is consistent with the previously observed result that cassette exons occur more often in smaller exons than larger ones, in both humans and *Drosophila* (Koralewski and Krutovsky 2011).

**Table 1:** Different types of Alternative Splicing in honey bee transcriptomes of two tissues

Tissue	Fat Body			Brain		
AS event	Number	Exons involved *	Total number of honey bee genes undergoing AS	Number	Exons involved *	Total number of honey bee genes undergoing AS
**Intron Retention (IR)**	11103	22204	3466 (22.6%)	8486	16972	2886 (18.8%)
**Cassette Exon (CE)**	1520	1520	1139 (7.4%)	1525	1525	1067 (7%)
**Alternative Donor Site**	2846	2245	1679 (11%)	2182	2008	1531 (10%)
**Alternative Acceptor Site**	4073	3624	2244 (14.7%)	3971	3322	2102 (13.7%)
**Alternative First Exon**	689	584	584 (3.8%)	308	281	281 (1.8%)
**Alternative Last Exon**	589	530	530 (3.5%)	545	402	402 (2.6%)

Alternative Exon Boundaries (AEB) includes Alternative Donor Site and Alternative Acceptor Site. Alternative Terminal Exons (ATE) includes Alternative First Exon and Alternative Last Exon. (* For retained introns, two flanking exons are counted as ‘involved’ exons.)

#### Intron retention:

Out of 69,323 honey bee introns in 13,407 multi-exon genes, we identified 11,103 (16%) IRs in 3,466 genes in fat body and 8,486 (12.2%) IRs in 2,886 genes in brain. We define an intron as a retained intron if each base of the intron has >5x coverage from our RNA-seq alignments. The average size of IRs was 716 bp in fat body and 1,174 bp in brain, smaller than that of the average honey bee introns (1,390 bp). This result is similar to IRs observed in *Drosophila* (Khodor *et al.* 2011).

#### Alternative exon boundaries and alternative terminal exons:

TrueSight detected 2,486 (2.9%) alternative 5′ sites in 1,679 genes in fat body and 2,182 (2.6%) alternative 5′ sites in 1,531 genes in brain. There were 4,073 (4.8%) alternative 3′ sites in 2,244 genes in fat body and 3,671 (4.3%) alternative 3′ sites in 2,102 genes in brain. In the fat body, ca. 4% of the genes in the honey bee genome have AFEs and 3.5% genes have ALEs; in brain the values are 2% and 3%, respectively. AEB seems to be the most common splicing pattern in honey bee and is consistent with observations made in *Drosophila* (Daines *et al.* 2011). 14 AFEs in fat body and 12 AFEs in brain have 3 bp displacements. 3 bp displacements in alternatively spliced transcripts involve addition or removal of 3 bps in the transcript start site or the end, which could alter gene functions. This was also observed in AFEs in *Drosophila*, which was generated through AS and alternative promoter usage with a few having the capacity to alter protein coding (Hanke and Storti 1988).

### Quantitative analysis of splice sites for different AS patterns

**Figure 1:**
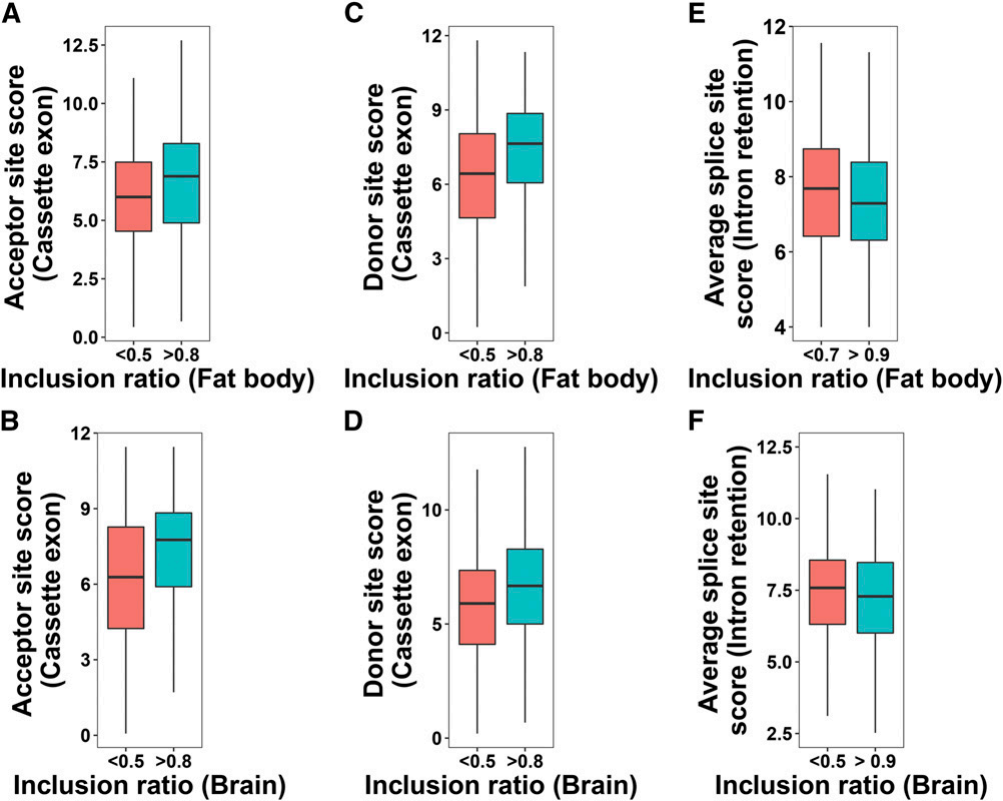
(A-D) Relationship between inclusion ratio and Donor/Acceptor site scores in cassette exons in honey bee fat body and brain. (E-F) Relationship between inclusion ratio and average splice site score in retained introns in fat body and brain. P-values from the Mann-Whitney-Wilcoxon tests for the two inclusion ratio categories: A = 0.0004476, B = 2.762e-06, C = 2.179e-06, D = 5.736e-05, E = 0.002843, F = 0.1034.

The strength of splice sites was assessed in CE and IR events by computing their donor and acceptor splice site scores. Acceptor and donor site scores were then plotted for different inclusion ratio categories in CEs and IRs. Here, inclusion ratio of an exon, for CE events, denotes the proportion of exons that span the exon skipping region based on the number of RNA-Seq reads mapped to that exon region. For IR events, inclusion ratio is calculated for introns. CEs with large inclusion ratios (>0.8) have stronger acceptor and donor sites than lower inclusion ratios (Figure 1A-D). IRs with large inclusion ratios on the other hand had weaker average splice site scores compared to lower inclusion ratio categories (Figure 1E-F). This observation is consistent with what was previously found in *Drosophila* (Khodor *et al.* 2011).

**Figure 2:**
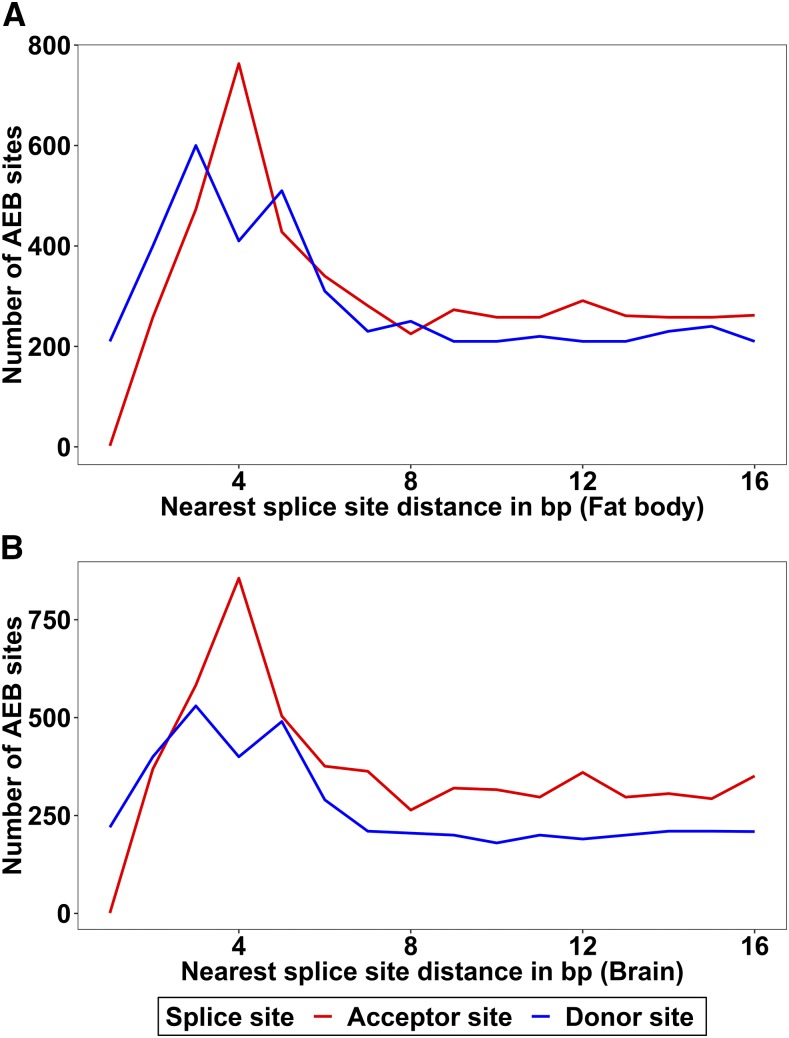
Distances of alternative 5′/3′ splice sites to the nearest splice sites are plotted for both donor and acceptor sites. A: Number of AEB sites for fat body, B: Number of AEB sites for brain. 4 bp and 3 bp gap dominates the alternative acceptor sites. The alternative donor sites show a dominance of 3 bp and 5 bp gap.

32% of AEB splice sites in fat body and 30.9% of AEB splice sites in brain were enriched in multiple of 3bp displacements, which usually preserve the reading frame of mRNAs. The alternative donor sites showed a dominance of 3 bp and 5 bp gap. This was found in both brain and fat body transcriptomes (Figure 2). Additionally, by comparing the motifs of 3/4/5 bp displaced AEB of both donor and acceptor sites, we found conservation patterns in frequencies of nucleotides at two proximal AS sites (Figure S3, Figure S4).

**Figure 3:**
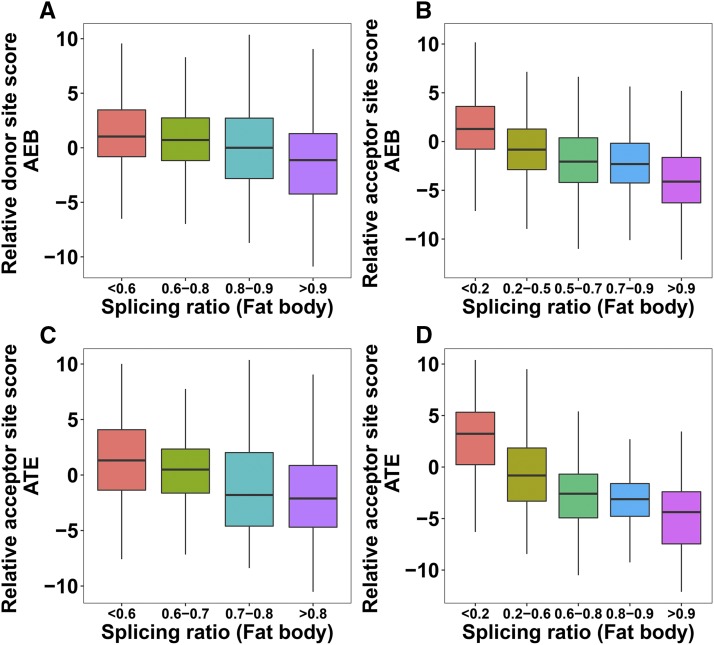
(A-B) Impact of relative splice site score (major - minor) on AEB splicing ratio (minor/major) in honey bee fat body. (C-D) Impact of relative splice site score (major - minor) on ATE splicing ratio (minor/major) in fat body. P-values from the Mann-Whitney-Wilcoxon tests between the first and the last splicing ratio categories: A = 2.2e-16, B = 2.2e-16, C = 8.189e-14, D = 2.2e-16.

It has been previously observed that expression ratios of isoforms are largely determined by competitiveness of nearby alternative splice sites (Xia *et al.* 2006; Yu *et al.* 2008). When comparing the relative splice site scores (major *vs.* minor) for both alternative donor and acceptor sites, we found that the expression ratio of the two isoforms (minor/major) goes up when the major splice site score goes down. This trend is clearer in fat body than brain but is observed in both (Figure 3A-B, Figure S5). Similar trends are also found in AFEs and ALEs (Figure 3C-D, Figure S5).

### Cross-species conservation of AS genes between honey bee and Drosophila

**Figure 4:**
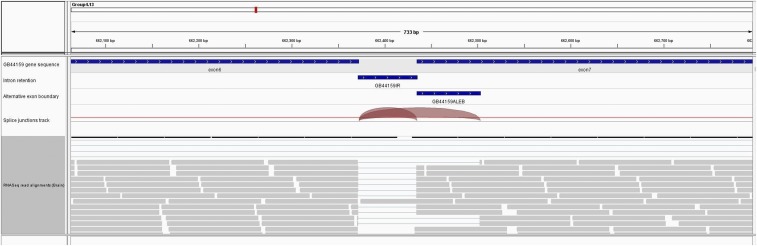
Alternatively spliced transcripts identified in *A. melifera* for the Dscam gene. Aligned reads are shown here in IGV genome browser (Thorvaldsdóttir *et al.* 2013). First track provides a zoomed in view of GB44159 (Dscam) gene in the honeybee (Amel 4.5) genome between exon 6 and 7 that has the AS event. Track 2 and 3 indicate the region corresponding to intron retention event and alternative left exon boundary respectively. Track 4 shows the splice junction spanning the AS events. Track 5 shows RNA-seq reads in honeybee brain mapped to this region.

More than 50% of AS genes (2,372 in fat body and 2,252 in brain) in honey bee have orthologs in *Drosophila* (Fig S6) and more than 50% of these genes (1,538 in fat body and 1,530 in brain) show AS in *Drosophila* as well (Table S4) (Brown *et al.* 2014). From all AS exon events, 448 orthologous genes had the same number of AS events in both *Drosophila* and honey bee, which is about 30% of the orthologous AS genes in *Drosophila*. We could not specifically conclude if these AS events are generally orthologous events or give rise to the same protein isoforms. Some of these genes are enriched for neuron development, sexual reproduction, and some basic cellular processes (Table S5). Interestingly, on visualizing the aligned reads in brain in the IGV genome browser (Thorvaldsdóttir *et al.* 2013), we found that the *Dscam* gene ortholog in honey bee (GB44159) undergoes alternative splicing as well and specifically shows an IR and an AEB in brain with high confidence (Figure 4). Previously, it has been shown that *AbsCAM* a Dscam family member in honey bee undergoes age specific alternative splicing in influencing neuronal wiring during development (Funada *et al.* 2007). This could provide potential insight into the conservation of the gene Dscam in neuronal development in terms of AS and how the AS forms could possibly affect the connectome in different castes.

### Connection between DNA methylation and alternative splicing

**Figure 5:**
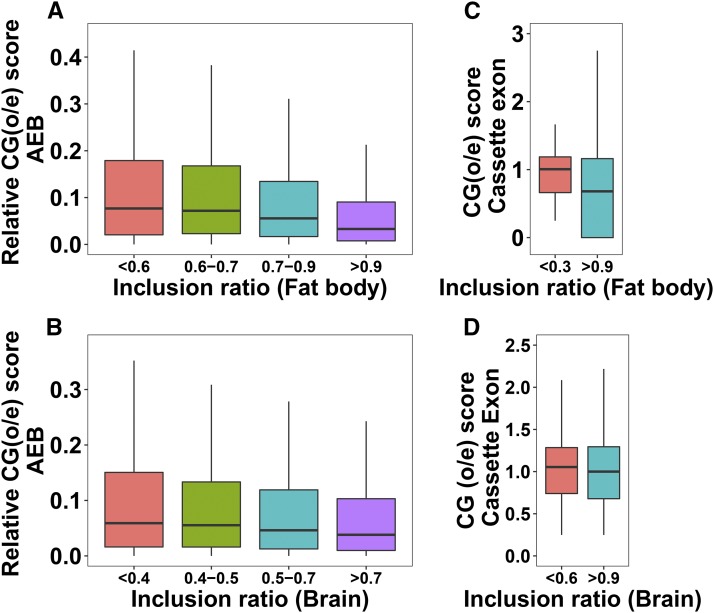
(A-B) Relationship between relative CG scores (difference in CG scores of alternative exon boundaries) and AEB inclusion ratio. (C-D) Relationship between CG score of cassette exon and their inclusion ratio. P-values from the Mann-Whitney-Wilcoxon tests for the first and the last inclusion ratio categories: A = 2.2e16, B = 1.427e-08, C = 0.01494, D = 0.2811.

Gene body methylation has been previously shown to play important roles in regulating AS (Flores *et al.* 2012; Li-Byarlay *et al.* 2013). We analyzed the correlation between methylation and splicing in terms of CpG observed-to-expected ratio (o/e) values for all AS exons. CpG (o/e) is a metric measuring the extent of DNA methylation (see Methods); a small or large CpG (o/e) value indicates hyper-methylation or hypo-methylation. To study the relationship between methylation levels in AEB regions and the inclusion ratio, the median of relative CpG (o/e) values for AEB regions was plotted for AEB inclusion ratio categories in fat body (Figure 5A) and brain (Figure 5B). Here the relative CpG score is the absolute value of the difference between CpG (o/e) of AEB exons. The relative CpG score was used here because this would denote the change in CpG score of the exon due to presence or absence of the AEB region. The lower the inclusion ratio of the AEB exon, the lower was the relative CpG score, which indicates that alternative exon boundaries that are not included often tend to be hyper-methylated. For CE and IR events, the median CpG score of the exons or introns having a certain inclusion ratio threshold was used as a metric to denote methylation levels. When comparing the median CpG (o/e) values for certain cassette exon (CE) inclusion ratio categories, we observed that CpG (o/e) values were lower (or higher) with CEs having higher (or lower) inclusion ratios in fat body (Figure 5C) and brain (Figure 5D). Therefore, more frequently included CEs might suggest higher methylation levels than rarely included CEs, in both tissues.

The median CpG (o/e) level in retained AS introns was 0.5 in fat body and 0.8 in brain, respectively. This is significantly lower than the CpG (o/e) in the set of all honey bee introns (1.06) (two-tailed *t*-test p-value < e^-10^), indicating higher methylation levels in retained introns. Knocking down DNA methyl transferase 3 (dnmt3) (Li-Byarlay *et al.* 2013) was shown to cause diverse and widespread changes in alternative splicing. CE events specifically showed increased exon skipping with decreased DNA methylation. Our results here support earlier evidence indicating that AS patterns are influenced by DNA methylation and provide new insights on the relationship between DNA methylation and all other splicing patterns (Flores *et al.* 2012; Li-Byarlay *et al.* 2013).

### Gene body methylation regulates tissue-specific splicing

We next used published Bisulfite sequencing (BS-seq) data in fat body (Li-Byarlay *et al.* 2013) to analyze the role of DNA methylation in tissue-specific splicing. We found that 3,830 (20%) AS events in fat body belonged to un-methylated regions, *i.e.*, CG pairs in these regions did not have methylated Cs and had an average CG score of 0.92 (Table S6). 2766 (15%) AS events in fat body on the other hand belonged to methylated regions, *i.e.*, CG pairs in these regions had methylated Cs and had an average CG score of 0.55. Moreover, 491 (12.1%) AS genes having *Drosophila* orthologs with AS belong to unmethylated regions whereas 361 (8.9%) AS genes with *Drosophila* orthologs with AS belong to methylated regions. Although there is high conservation of AS genes between *Drosophila* and honey bee, DNA methylation is not the main mechanism regulating AS in *Drosophila* (Urieli-Shoval *et al.* 1982; Lyko *et al.* 2000; Kunert *et al.* 2003). This observation suggests that changes in methylation levels within the gene body could be one of the possible regulatory mechanisms for tissue-specific splicing in honey bee, unlike *Drosophila*. BS-seq data corresponding to RNASeq data in brain that was used in this study was unavailable. Hence, we could not draw specific conclusions on methylation levels in Brain specific AS events.

### Functional analysis of alternatively spliced genes

Gene Ontology analysis revealed that common genes that undergo AS in both tissue types are specifically enriched in housekeeping functions related to cell differentiation, regulation of signaling and response to stimulus (Table S7). More than 50% of these common genes have AS events that are tissue specific (Table S7). Common AS genes that have tissue-specific AS events were involved in some biological pathways based on previous literature and these pathways have been mentioned below (Foret *et al.* 2009). There were more than 20 genes with tissue-specific AS events in both tissues in specific KEGG pathway categories responsible for metabolism, *i.e.*, insulin/TOR signaling, oxidative phosphorylation (Table S7). There were 31 genes in fat body and 28 genes in brain that are involved in the spliceosome pathway (Table S7). These results highlight the possibility that splicing is widespread in genes that have housekeeping functions and that isoforms could be generated for these general functions in a tissue-specific manner. These results are also consistent with the previous results about AS genes in honey bee, *i.e.*, ubiquitously expressed genes often lead to production of tissue-specific isoforms (Foret *et al.* 2012).

Among the various tissue-specific functional categories, AS genes specific to fat body and brain are predominantly enriched in protein metabolism and neuron development, respectively (Table S8). We also observed 33 genes in brain that include some TFs and TF targets in the brain transcriptional regulatory network (Table S8). The fact that some TFs in this network undergo alternative splicing in brain may provide new perspectives to the gene regulatory mechanisms in different tissue types.

## Discussion

Our analyses of RNA-seq datasets from two different tissue types enabled us to identify tissue-specific AS patterns in the honey bee. IRs and AEBs dominate among all splicing patterns identified by TrueSight. Previous research has shown an expansion of protein families encoded by certain genes in honey bees due to alternative exons, and these genes are known to play a key role in neurological disorders, sexual differentiation, and reproduction (Beye *et al.* 2003; Jones *et al.* 2006; Jarosch *et al.* 2011). This is consistent with our observations of the frequency of AEBs. Consistent with these studies, Cassette exons and retained introns in fat body and brain are smaller in length than the average size of honey bee exons and introns. This observation is consistent with the trends in *Drosophila* (Khodor *et al.* 2011; Koralewski and Krutovsky 2011). Most invertebrates have much smaller introns as compared to vertebrates, which results in IR events due to a failure in recognizing splice sites (Talerico and Berget 1994). Although the intron sizes in invertebrates are smaller than those observed in vertebrates, a similar trend was also observed for IRs in vertebrates (Gelfman *et al.* 2012).

We reported correlations between the extent of AS and parameters characterizing AS patterns, namely splice site strength, exon-intron structure, and methylation patterns for every AS event. Analyzing the strength of splice sites for splicing patterns shows that CEs with large inclusion ratios (>0.8) have stronger acceptor sites and donor sites. One explanation is that CEs with strong donor and acceptor sites would splice the flanking introns thus are included more frequently in the transcript. IRs with higher inclusion ratios had weaker splice sites. According to the intron definition model, which is prevalent in invertebrates, the strong donor or acceptor sites flanking an intron would be recognized to splice introns out in most transcripts (Talerico and Berget 1994).

Most AEB splice sites are enriched in multiples of three displacements, which preserve the reading frame of the mRNAs. The conserved patterns of 3 bp displacements might give us new perspective on the predominant splicing patterns that contribute to protein diversity for many uncharacterized genes in the honey bee genome, as AEBs are the most common form of AS events observed in this species. In addition, the connection between gene body DNA methylation and AS patterns, especially tissue specific AS patterns, further confirms the role of methylation in AS regulation and the distinct gene regulatory functions of those AS genes (Li-Byarlay *et al.* 2013; Foret *et al.* 2012).

High throughput RNA-seq data were analyzed with TrueSight to reveal various splicing patterns observed in brain and fat body. This provides us with information on splicing-specific regulation of honey bee genes in brain and fat body, as well as possible methylation driven AS not observed in *Drosophila* in spite of cross species conservation. Although our analysis of AS patterns was limited to only one fat body and one brain RNA-seq study, this nevertheless provides preliminary observations that could be useful for unraveling unknown gene regulatory mechanisms through AS. Future species-specific transcriptomic approaches could build upon the current set of AS dataset provided in this study. Understanding the mechanisms of these AS patterns in honey bee, a model organism representing behavioral plasticity, may eventually shed light on the molecular regulation of behavioral phenotypes.
